# Modified small vessel disease score as the top predictor of stroke outcome after thrombectomy: a CT-based machine learning study

**DOI:** 10.3389/fneur.2026.1622586

**Published:** 2026-06-23

**Authors:** Thiago Oscar Goulart, Rui Kleber do Vale Martins-Filho, Millene Rodrigues Camilo, Daniel Giansante Abud, Octávio Marques Pontes-Neto

**Affiliations:** 1 Department of Epidemiology, Harvard T.H. Chan School of Public Health, Boston, MA, United States; 2 Department of Neuroscience and Behavioral Sciences, Ribeirão Preto Medical School, University of São Paulo, Ribeirão Preto, São Paulo, Brazil

**Keywords:** stroke, ischemic stroke (IS), thrombectomy, reperfusion therapies, low and middle income countries, machine learning, artificial intelligence, cerebral small vessel disease

## Abstract

**Background:**

Mechanical thrombectomy (MT) improves outcomes in ischemic stroke (IS) due to large vessel occlusion (LVO), but ~50% of patients fail to achieve functional independence.

**Objectives:**

We investigated whether cerebral small vessel disease (cSVD), assessed by the modified Small Vessel Disease (mSVD) score and Brain Frailty Score (BFS), outperforms individual CT markers in predicting 90-day outcomes after MT.

**Design:**

Prospective cohort with retrospective analysis.

**Methods:**

We included 351 patients with anterior circulation LVO treated with MT. Admission CT was used to score cSVD markers (leukoaraiosis, atrophy, lacunes) and compute mSVD and BFS. Eight logistic regression models and a Random Forest algorithm were used to predict poor outcome [modified Rankin Scale (mRS) 3–6]. Model performance was evaluated using AUC-ROC and compared via DeLong tests.

**Results:**

Poor outcomes were associated with older age, higher NIHSS, systolic blood pressure, glycemia, and more severe leukoaraiosis and atrophy. Severe mSVD (score = 3) independently predicted poor outcomes (OR = 3.267; CI: 1.731–6.168; *p* = 0.009). mSVD outperformed BFS and individual CT markers (AUC = 0.904 vs. 0.889/0.898; DeLong *p* < 0.05) and ranked as the top predictor in Random Forest (importance = 42.05). Treatment efficacy declined with increasing mSVD: the probability of a favorable outcome was 15.53% and poor outcome was 84.47% for mSVD = 3, compared to 89.23% and 10.77%, respectively, for mSVD = 0. A secondary model incorporating 24h NIHSS and hemorrhagic transformation improved discrimination (AUC = 0.954), but mSVD remained a key independent predictor.

**Conclusions:**

In this prospective study in a middle-income country, mSVD score was the strongest predictor of post-thrombectomy outcome, outperforming BFS and isolated imaging markers. While cSVD does not contraindicate MT, it reflects reduced cerebrovascular resilience. Integrating mSVD into baseline CT evaluation may enhance risk stratification and treatment guidance.

## Introduction

Mechanical thrombectomy (MT) has been firmly established as a safe and effective treatment for acute ischemic stroke (AIS) caused by large vessel occlusion (LVO). Landmark studies such as MR CLEAN, ESCAPE, REVASCAT, SWIFT PRIME, and EXTEND-IA, later aggregated in the HERMES meta-analysis, demonstrated the efficacy of MT within a 6-h time window (1–6). Subsequent trials expanded its indications, including posterior circulation strokes (7), extended window, and cases with large ischemic cores (8).

Despite these advances, a significant proportion of patients—approximately 50%, according to Nie et al. (9)—do not achieve favorable outcomes (mRS ≤ 2) despite successful recanalization. Molina et al. (10) had termed this phenomenon “futile recanalization” (FR), though the appropriateness of this term has been questioned given potential functional gains even in mRS 3–4.

While many predictors of poor outcomes have been identified, including age, ASPECTS, collateral status, admission hyperglycemia, and NIHSS (11), recent evidence suggests that brain frailty (BFS) and small vessel disease (SVD) scores may play a crucial role in this context. These scores integrate radiological markers such as leukoaraiosis, brain atrophy, and chronic infarcts to quantify cerebrovascular burden and resilience. Appleton et al. (12) demonstrated their utility in predicting outcomes in IS patients, while Pedraza et al. (13) found that brain atrophy independently predicted FR and synergized with age and infarct volume to affect MT outcomes.

These findings underscore the need for a comprehensive understanding of multimorbidity's impact on stroke outcomes, especially in populations undergoing MT for LVO.

## Objectives

This study aims to evaluate the influence of BFS and mSVD in the outcomes of patients with AIS due to LVO of anterior circulation undergoing MT.

## Methodology

### Populations and study design

This study analyzed data from 351 patients with anterior circulation LVO AIS treated with MT, drawn from a prospective cohort at a tertiary hospital in a middle-income country, admitted from January 2018 to December 2022.

### Data sources and ethical considerations

Data were collected from a prospective registry approved by the Institutional Ethics Committee (CAAE 19624213.6.0000.5440). Clinical and radiological data were recorded systematically, ensuring compliance with ethical guidelines.

### Data collection

Clinical data included demographic information (age, gender, and ethnicity categorized as Caucasian or non-Caucasian), pre-stroke modified Rankin Scale (mRS), prior anticoagulant/antiplatelet use, and history of stroke or transient ischemic attack (TIA). Comorbidities were documented, including hypertension, diabetes mellitus, dyslipidemia, obesity, cardiac disease (classified according to ASCOD criteria), and smoking or alcohol use. At admission, blood pressure, blood glucose levels, and NIH Stroke Scale (NIHSS) scores were recorded. Functional outcomes (mRS) were assessed 90 days post-stroke.

Laboratory data included hemoglobin A1c (HbA1c), lipid profile, and serum creatinine, with contrast-induced nephropathy (CIN) defined as an increase in serum creatinine of ≥0.5 mg/dl (or ≥25% from baseline) within 48–72 h of contrast media administration, calculated using the CKD-EPI formula. Cocaine metabolite (benzoylecgonine) was assessed when available. The stroke etiology was evaluated based on ASCOD criteria.

Radiological data were derived from baseline and follow-up CT scans. Baseline assessments included ASPECTS, leukoaraiosis, collateral score, brain atrophy, and prior ischemic lesions. Follow-up CT scans (24–72 h post-MT) were used to differentiate contrast extravasation (CE) from HT and further categorized HT subtypes (HI-1/HI-2, PH-1/PH-2), according to ECASS-3 criteria.

Recanalization therapy variables included documentation of thrombolysis (door-to-needle time) and thrombectomy (occlusion site, time to puncture, time to reperfusion, mTICI scale, and angioplasty). Recanalization was defined as mTICI 2b-3.

### Imaging analysis of cerebral small vessel disease

CT scans were independently reviewed by a vascular neurologist blinded to clinical data.

### Assessment of small vessel disease markers

- White matter hypodensities (leukoaraiosis): evaluated on three standardized axial slices: choroid plexus, the middle cella, and the centrum semiovale (12). Leukoaraiosis was graded as 0 (absent), 1 (mild/moderate), or 2 (severe), based on the extent and intensity of hypodensity (Supplementary Figures 1, 2).
- Atrophy: assessing central atrophy (ventricular enlargement) and cortical atrophy (sulcal prominence). The highest score between central and cortical atrophy was used, with grades defined as 0 (absent), 1 (mild/moderate), or 2 (severe; Supplementary Figure 3).
- Lacunar infarcts: identified as hypodense lesions < 15 mm in diameter in deep gray matter or white matter, consistent with prior small vessel ischemic injury.

### Score calculations

The modified small vessel disease (mSVD) score, which ranges from 0 to 3, was calculated by assigning one point for each of the following findings on baseline CT:

- Severe leukoaraiosis (graded as 2).
- Severe atrophy (graded as 2).
- Presence of lacunar infarcts.

The brain frailty score (BFS) included a broader evaluation by scoring:

- Leukoaraiosis graded as 1 (mild/moderate) or 2 (severe).
- Atrophy graded as 1 (mild/moderate) or 2 (severe).
- Old vascular lesions/infarcts.

Each marker contributed one point, resulting in BFS scores ranging from 0 to 3. The BFS allowed for the inclusion of less severe markers of small vessel disease, capturing a wider spectrum of cerebrovascular frailty. Both scores were validated by Appleton et al. (12).

### Statistical analysis

Predictors of functional dependence (mRS > 2) were assessed using a systematic approach that included univariate and multivariable analyses. The univariate analysis was conducted first, calculating the chi-square test for categorical variables and appropriate parametric or non-parametric tests (e.g., *t*-test or Mann-Whitney *U*-test) for numerical variables to determine *p*-values. Variables with clinical relevance and a *p*-value < 0.1 in the univariate analysis were selected for inclusion in the initial multivariable model.

A backward stepwise regression approach was then applied to construct the most parsimonious multivariable logistic regression model. The Akaike Information Criterion (AIC) was used as the selection criterion to balance model complexity and goodness of fit. Multicollinearity was assessed using the Variance Inflation Factor (VIF), and variables with VIF > 5 were excluded to ensure model stability. Interaction terms (e.g., between mSVD and age) and polynomial terms for age, chronic kidney disease (CKD), and cerebral small vessel disease (cSVD) markers (mSVD and BFS scores) were tested but did not achieve statistical significance. As a result, models with a linear approach were retained.

To address specific research questions, eight additional models were built to evaluate different approaches to key variables. Model 1 was derived from the backward stepwise regression process and included statistically significant variables with low multicollinearity. In Model 2, mSVD was replaced by BFS due to a VIF > 5 when both were included simultaneously. Model 3 incorporated two additional clinically relevant variables to explore their contributions. Model 4 again substituted BFS for mSVD to address collinearity concerns. Model 5 introduced a composite score combining mSVD and BFS, aiming to balance the overestimation bias of BFS and the underestimation bias of mSVD. Model 6 replaced composite scores with individual markers of cerebral small vessel disease, including leukoaraiosis, atrophy, and prior lacunes. Model 7 dichotomized leukoaraiosis into severe vs. non-severe and included other simplified variables to enhance interpretability. Finally, Model 8 was developed to incorporate variables collected after 24 h to develop a predictive model with data of 24 h after admission, substituting NIHSS (Admission) with NIHSS (24 h) and including hemorrhagic transformation (ECASS 1–4). Model discrimination was assessed using the area under the receiver operating characteristic curve (AUC-ROC).

#### Model calibration

Calibration of the logistic regression models was assessed using the Hosmer-Lemeshow goodness-of-fit test, which compares observed and predicted event rates across deciles of predicted probability. A *p*-value greater than 0.05 indicates good calibration. In addition, calibration plots were generated to visually assess agreement between predicted and observed outcomes. Overall model performance was also evaluated using the Brier score, which quantifies the accuracy of probabilistic predictions (lower scores indicate better performance). To mitigate overfitting in logistic regression models, we selected clinically meaningful predictors *a priori* and evaluated model calibration using the Hosmer-Lemeshow test and Brier score.

The Random Forest model was, also, used to predict dependency (mRS: 3–6) at 90 days post-ictus. The dataset was split into training (80%) and test (20%) sets, ensuring stratified sampling to preserve class proportions. Predictor variables were selected using LASSO regression and included NIHSS (Admission), SBP (Admission), glycemia, delta CKD, BFS, mSVD, and dependency. The dependent variable was converted to a factor to ensure proper model interpretation.

Overfitting was minimized by performing five-fold cross-validation during hyperparameter tuning, which involved testing combinations of mtry, ntree, and nodesize. The configuration yielding the highest accuracy was selected. Variable importance was assessed using the Mean Decrease in Accuracy. The following ranges were tested:

- mtry: 2, 3, 4
- ntree: 100, 200, 300, 500, 1,000
- nodesize: 1, 5, 10

Performance metrics, including accuracy, were evaluated for each hyperparameter combination to determine the optimal configuration. Variable importance was assessed using the Mean Decrease in Accuracy, ranking predictors based on their impact on the model's performance.

The probabilities of treatment efficacy (favorable outcome) and treatment failure (poor outcome) at 90 days post-thrombectomy were calculated for individual mSVD scores (0, 1, 2, 3) and grouped scores (0–1 vs. 2–3). These metrics quantified the relative benefits and risks across varying levels of small vessel disease burden.

## Results

### Patient characteristics

The study included 351 patients with AIS due to LVO of AC MT. The mean age was 67.0 ± 6.98 years, with 48.3% male patients (Supplementary Table 1). Most patients identified as Caucasian (78.0%), followed by 12.9% “Parda,” 8.0% Black, and 1.1% Asian. Pre-stroke functional independence (mRS = 0) was reported in 74.3%, while 11.1% had a pre-stroke mRS of 1.

Hypertension was the most prevalent comorbidity (78.9%), followed by dyslipidemia (53.1%), diabetes mellitus (26.3%), and cardiopathy (48.3%). A history of prior stroke or TIA was documented in 8.9%. Tobacco use was reported by 31.4% of patients, while alcohol consumption was noted in 19.1%. Cocaine use was infrequent, observed in 1.1% of cases.

The median ASPECTS score was 7.72 ± 1.68, with severe brain atrophy identified in 34.0% of patients and severe leukoaraiosis in 52.3%. BFS scores of 3, reflecting significant cerebral frailty, were observed in 68.6%, and mSVD scores of 3 were noted in 29.4%. Poor collateral circulation (Degree 0–1) was present in 68.9% of patients.

Mechanical thrombectomy achieved successful recanalization in 92.3% of cases. The median time from ictus to recanalization was 368.5 min, and angioplasty was performed in 25.1% of patients.

The mean admission glycemia was 121.8 ± 16.3 mg/dl. Contrast-induced nephropathy occurred in 12.0% of patients. Symptomatic hemorrhagic transformation (SHT) was observed 7.7%.

The median NIHSS score at admission was 16.67 ± 3.68, remaining stable at 17 during the first 24 h post-MT. Functional independence (mRS ≤ 2) at 90 days was achieved in a subset of patients, though the high prevalence of severe SVD markers highlights the challenges of achieving favorable outcomes in this cohort. Associations between cerebral frailty and dependency outcomes will be explored further in subsequent analyses.

Several variables were associated with poor outcomes (mRS: 3–6) at 90 days post-stroke (Supplementary Table 2). Patients with mRS 3–6 had a significantly higher mean age compared to those with mRS 0–2 (68.14 ± 12.44 vs. 61.86 ± 14.48 years, *p* < 0.001). Significant differences were observed for pre-stroke mRS (*p* = 0.018), with a higher proportion of patients with dependency at baseline among those with worse outcomes.

Diabetes mellitus (33.77 vs. 12.30%, *p* < 0.001), tobacco use (35.96 vs. 22.95%, *p* = 0.017), higher mean glucose (143.32 ± 62.50 vs. 117.57 ± 39.84 mg/dl, *p* < 0.001) and creatinine levels (1.33 ± 0.99 vs. 0.95 ± 0.22 mg/dl, *p* < 0.001) were more prevalent in the mRS 3–6 group. Lower ASPECTS (7.61 ± 1.75 vs. 7.92 ± 1.53, *p* = 0.038), worse collateral circulation scores (*p* < 0.001), and higher prevalence of severe leukoaraiosis (69.74 vs. 19.51%, *p* < 0.001), were strongly associated with poor outcomes.

### Multivariable logistic regression models

Eight multivariable logistic regression models were tested to assess the predictive value of mSVD, BFS, individual CT markers, and clinical variables.

**Figure 1:**
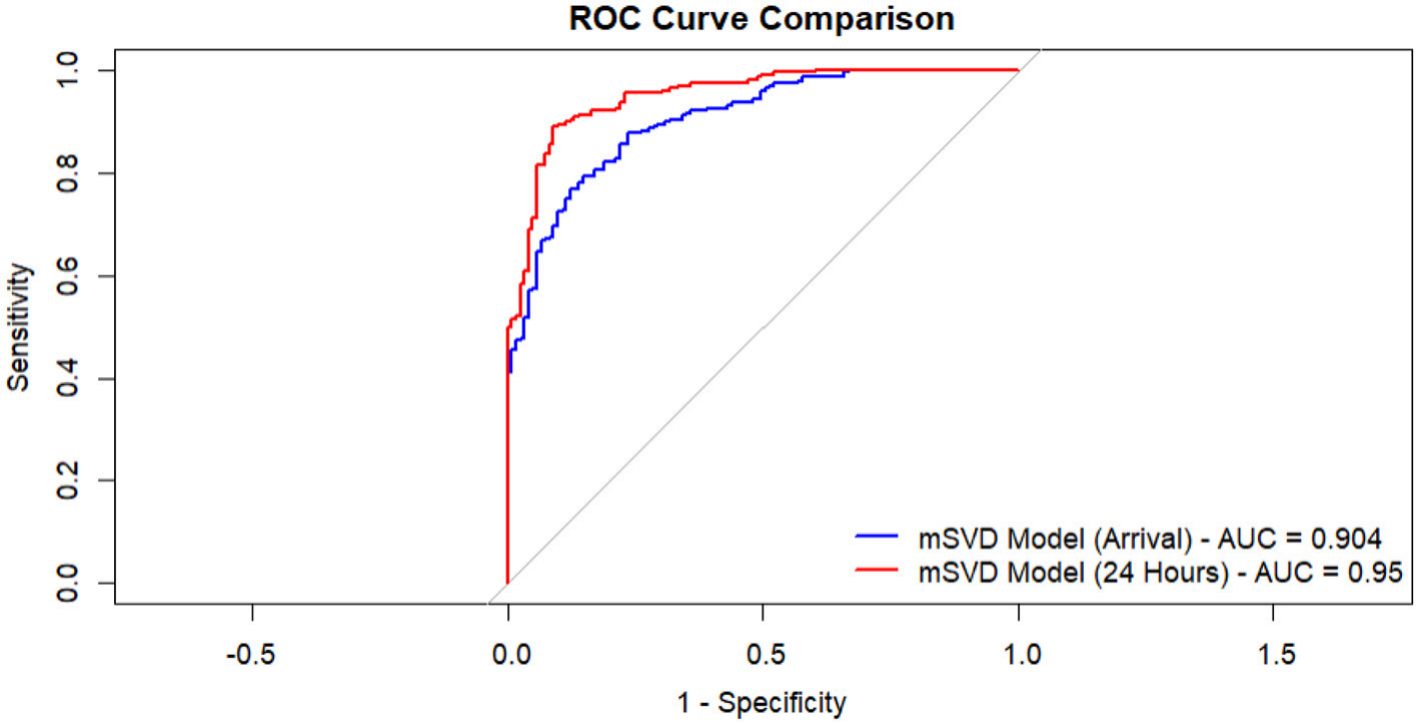
ROC curve comparison of two logistic regression models: the baseline model using arrival data (AUC = 0.904) and the extended model incorporating 24-h variables (NIHSS and hemorrhagic transformation; AUC = 0.950). The curves illustrate improved discrimination with the inclusion of early post-treatment information.

Model 1 included mSVD as a key predictor, revealing a dose-response relationship with increasing scores (OR for Score 3: 39.760, 95% CI: 19.113–82.673, *p* < 0.001). Other significant predictors were NIHSS at admission (OR: 1.087, *p* < 0.001), glucose (OR: 1.013, *p* < 0.001), and recanalization (OR: 0.119, *p* = 0.023). This model demonstrated excellent discrimination (AUC = 0.904) (Figure 1).

Model 2 substituted BFS for mSVD and showed similarly significant associations, with BFS Score 3 having an OR of 46.466 (95% CI: 2.32–6.35, *p* < 0.001). The AUC was slightly lower at 0.889.

Model 3 introduced clinically relevant variables, including obesity and age, alongside mSVD. Obesity demonstrated to be a protective factor (OR: 0.341, *p* = 0.005), which is consistent with the “Obesity Paradox,” which will be addressed in the discussion. NIHSS at admission (OR: 1.116, *p* < 0.001), systolic blood pressure (OR: 1.018, *p* = 0.002), and glucose levels (OR: 1.010, *p* = 0.021) retained their significance. mSVD (High) was strongly associated with lower odds of functional independence (OR: 10.56, *p* < 0.001). The AUC for this model was 0.889.

Model 4 reintroduced BFS along with additional variables. BFS (High) was strongly associated with lower odds of functional independence (OR: 13.8, *p* < 0.001). NIHSS at admission (OR: 1.114, *p* < 0.001) and glucose levels (OR: 1.010, *p* = 0.027) remained significant, while obesity (OR: 0.406, *p* = 0.012) continued to demonstrate a protective effect. The AUC for this model was 0.861.

Model 5 used a composite score combining mSVD and BFS to address potential underestimation or overestimation of cSVD burden. Higher composite scores were strongly predictive of poor outcomes (OR for Score 6: 33.622, *p* < 0.001). AUC improved to 0.907, the highest among all models.

Model 6 included isolated markers of cSVD, such as leukoaraiosis, atrophy, and prior lesions. Severe leukoaraiosis (OR: 10.792, *p* < 0.001) and prior lesions (OR: 3.673, *p* = 0.017) were significant. The AUC was 0.898.

Model 7 simplified Model 6 by dichotomizing leukoaraiosis and excluding atrophy. Severe leukoaraiosis (OR: 3.485, *p* = 0.016) and lacunar infarcts (OR: 9.144, *p* < 0.001) remained significant predictors. The AUC was 0.887.

Model 8 achieved an AUC of 0.954 (Figure 1) and highlighted the significance of variables such as NIHSS (24 h; OR: 1.151, *p* < 0.001) and hemorrhagic transformation (OR: 1.865, *p* < 0.001). The mSVD categories remained significant predictors, with mSVD3 showing an OR of 3.267 (*p* = 0.009).

All logistic regression models showed good calibration, with Hosmer-Lemeshow test *p*-values ranging from 0.247 to 0.550, indicating no significant deviation between predicted and observed outcomes. The Brier score ranged from 0.129 to 0.139, demonstrating good overall prediction accuracy. Table 1 in Supplementary Datasheet summarizes the calibration results for the four main models, and Supplementary Figures 5 and 6 present the calibration curves for Models 1 and 8, respectively.

### Random forest

The optimal Random Forest configuration identified during hyperparameter tuning was mtry = 2, ntree = 1,000, and nodesize = 10. Using this configuration, the model achieved excellent performance, with an accuracy of 0.925 in the training set and 0.811 in the test set. The learning curve demonstrated high training accuracy (near 1.0) and slightly lower test accuracy, indicating good generalization with minimal overfitting.

**Figure 2:**
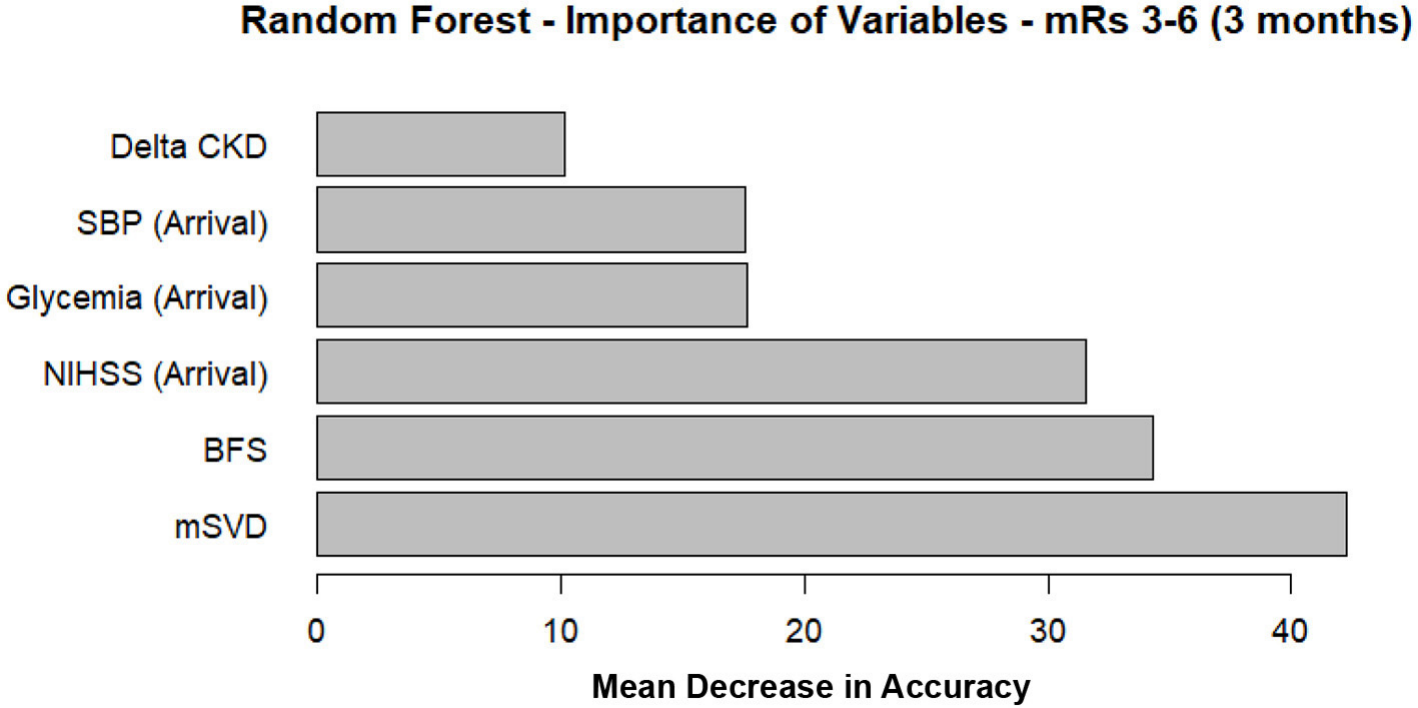
Random Forest variable importance plot for predicting functional dependence (modified Rankin Scale 3–6) at 90 days post-thrombectomy. Variable importance was measured using permutation-based *mean decrease in accuracy*, which quantifies the reduction in model performance when each variable is randomly permuted. Higher values indicate greater predictive relevance.

Variable importance analysis revealed the following rankings, based on the Mean Decrease in Accuracy (Figure 2):

1. mSVD (42.05)
2. BFS (30.97)
3. NIHSS (Arrival; 26.91)
4. SBP (Arrival; 17.10)
5. Delta CKD (12.87)
6. Glycemia (11.30)

The results highlight the dominant roles of mSVD and BFS in predicting poor functional outcomes, followed by NIHSS (Admission) and systemic factors such as SBP and glycemia.

### Probability of favorable and poor outcomes

**Figure 3:**
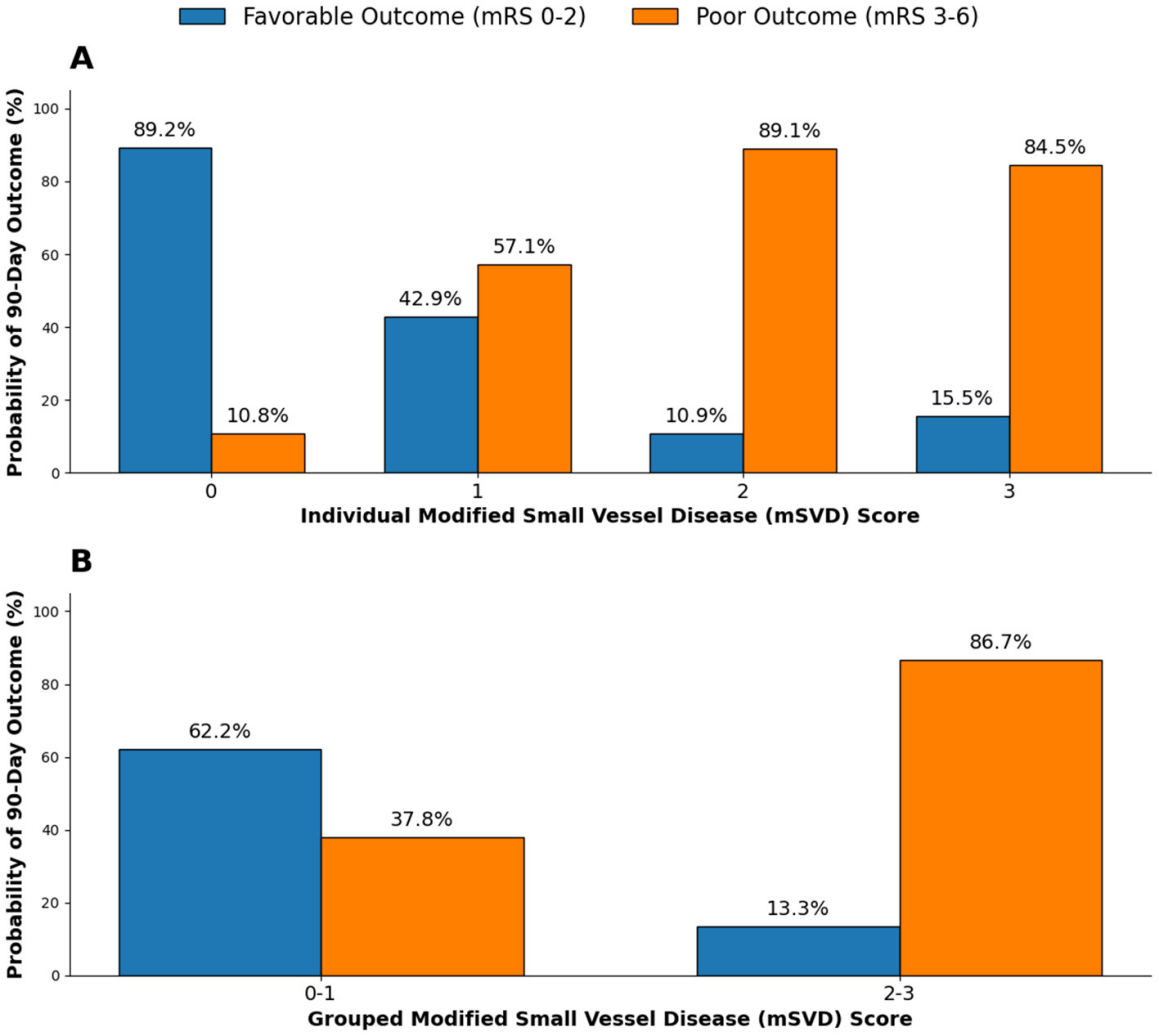
**(A)** Probability of Outcomes for Individual mSVD Scores (0, 1, 2, 3)—illustrates the variation in the probability of favorable and poor outcomes across individual modified Small Vessel Disease (mSVD) scores. **(B)** Probability of Outcomes for Grouped mSVD Scores (0–1 vs. 2–3)—demonstrates the differences in outcome probabilities when mSVD scores are grouped into lower (0–1) and higher (2–3) cerebrovascular disease burden categories.

The probabilities of treatment effectiveness (favorable outcome) and treatment failure (poor outcome) for both individual mSVD scores and grouped scores (0–1 and 2–3) are summarized below, as illustrated in Figure 3.

- mSVD = 0: Favorable Outcome = 89.23%, Poor Outcome = 10.77%.
- mSVD = 1: Favorable Outcome = 42.86%, Poor Outcome = 57.14%.
- mSVD = 2: Favorable Outcome = 10.87%, Poor Outcome = 89.13%.
- mSVD = 3: Favorable Outcome = 15.53%, Poor Outcome = 84.47%.
- mSVD = 0–1: Favorable Outcome = 62.18%, Poor Outcome = 37.82%.
- mSVD = 2–3: Favorable Outcome = 13.33%, Poor Outcome = 86.67%.

## Discussion

This study highlights the pivotal role of cSVD markers and composite scores (mSVD and BFS) in predicting poor functional outcomes (mRS 3–6) at 90 days post-thrombectomy for anterior circulation LVO. While age is a well-documented risk factor for poor stroke outcomes, it did not independently predict functional outcomes in any of our models. This suggests that brain resilience—reflected by markers such as leukoaraiosis, lacunar infarcts, and atrophy, as captured by BFS and mSVD—may have a greater impact on functional recovery than chronological age. These scores serve as indicators of overall brain health, where cumulative cerebrovascular injury plays a crucial role in determining poor outcomes following acute reperfusion therapies.

Notably, some models yielded very high odds ratios, particularly for mSVD and BFS scores of 3. These values reflect the strong association between severe cerebrovascular frailty and poor outcomes but must be interpreted with caution. The relatively smaller number of patients with extreme scores may contribute to wider confidence intervals and increased estimate variability. Furthermore, as logistic regression models the log-odds of the outcome, the resulting odds ratios are exponential in nature. This means that strong linear relationships in the log-odds scale can translate into disproportionately large ORs when exponentiated, especially for powerful predictors such as mSVD = 3. This behavior is expected in models with steep dose-response relationships and should not be misconstrued as model overfitting or instability.

While thrombectomy restores macrovascular blood flow, the impaired microcirculation in regions with extensive cSVD may limit tissue perfusion (14). This mismatch indicates that compromised small vessels can act as a constraint, compromising adequate reperfusion of ischemic territories even after successful large vessel recanalization. This observation reinforces the need to consider macro and microvascular health when evaluating thrombectomy outcomes.

Accordingly, analyzing the probabilities of favorable and poor outcomes based on mSVD scores offered valuable insights into the relationship between treatment efficacy and associated risks. For patients with minimal cSVD burden (mSVD = 0), the probability of a favorable outcome was remarkably high at 89.23%, indicating highly effective treatment and low associated risks. However, for patients with severe cSVD (mSVD = 3), the probability of a favorable outcome dropped substantially to 15.53%, and the probability of a poor outcome increased to 84.47%, indicating reduced efficacy and heightened risks. These findings suggest a progressive decline in the benefits of thrombectomy as cSVD burden increases, likely due to the inability of damaged microvasculature to support effective reperfusion and recovery.

Additionally, we must point out the high prevalence of severe cSVD markers observed in this cohort, which aligns with patterns typically seen in low- and middle-income countries (LMIC), where the prevention and management of classical cardiovascular risk factors, such as hypertension, diabetes, and dyslipidemia, are less robust compared to developed nations (15). This disparity reflects systemic challenges in access to healthcare, health education, and preventive strategies in resource-limited settings. Consequently, the “brain health” of populations in developed countries may be comparatively better due to more effective prevention and control of these risk factors. This difference in cerebrovascular burden could have a greater chance of influencing thrombectomy outcomes in LMIC, where compromised brain resilience may exacerbate the challenges of achieving favorable functional recovery. Addressing these disparities is critical to improving stroke care and outcomes globally, particularly in regions with the highest stroke burden.

Models 3 and 4 demonstrated an obesity paradox, with obesity showing a protective effect on functional outcomes (Model 3: OR 0.341, 95% CI: 0.17–0.70, *p* = 0.005; Model 4: OR 0.406, 95% CI: 0.20–0.83, *p* = 0.012). This finding aligns with prior studies, where the paradox persisted even after propensity score matching (16, 17). The persistence of this association, despite statistical adjustments, suggests that body composition, rather than BMI alone, may play a role, as older adults generally have lower lean mass, which could influence post-stroke recovery. Further research is needed to better understand this phenomenon.

From a socioeconomic perspective, the burden of stroke represents a critical challenge for public health policy, especially in LMIC such as Brazil, driven by a persistent trend of increasing incidence and escalating healthcare costs across all stroke subtypes (19–24). While the individual cost of care for hemorrhagic stroke is often Higher (19–22), hospitalization expenditures for ischemic stroke are particularly taxing due to their significantly higher volume and the long-term resources required for management (23).

Addressing this burden requires intervention at both extremes of the stroke care continuum. At one end, there must be a robust focus on primary prevention and the aggressive control of modifiable risk factors to preserve cerebrovascular resilience (24). At the opposite end of the spectrum, the survival and recovery of these patients depend on highly specialized neurocritical care. However, in low- and middle-income countries (LMICs), this acute end of care remains profoundly heterogeneous, a systemic disparity that directly compromises the outcomes of complex neurological emergencies (25).

In the acute setting, managing secondary stroke etiologies like atrial fibrillation introduces significant clinical complexity, as the necessity for anticoagulation frequently clashes with pre-existing markers of cerebrovascular vulnerability (26, 27). Structural lesions, such as lacunes, brain atrophy, and white matter hyperintensities (28), act as indicators of “Systemic Microvasculature Frailty,” imposing an additional burden that independently predicts poor functional recovery and a heightened susceptibility to post-procedural complications like contrast-associated acute kidney injury (29). Crucially, these markers of structural frailty significantly (30), alongside hemorrhagic infarct (31), amplify the risk of catastrophic bleeding and subsequent functional decline. Indeed, a recent subanalysis of RESILIENT trial, also showed that CT-based CSVD markers predict poor outcomes after thrombectomy (32).

This structural vulnerability does not exist in isolation; it is inextricably linked to broader cardiovascular and endothelial health. Systemic conditions such as a lower cardiac index and impaired aortic compliance directly reduce cerebral blood flow, which independently exacerbates WMH burden (33). To navigate this precarious balance between embolic prevention and bleeding risk, emerging biomarkers offer a promising approach to accurately quantify vascular frailty prior to treatment. For example, elevated syndecan-1 levels indicate profound endothelial glycocalyx damage (34), while identifying pre-treatment hemorrhagic infarction on MRI serves as a critical warning for reperfusion injury (33). Ultimately, integrating these novel biomarkers and cardiovascular indices with advanced artificial intelligence tools, such as those capable of distinguishing true hemorrhage from contrast extravasation (35, 36), will be essential for refining clinical decision-making in highly vulnerable patients

### Strengths

The study's strengths include its relatively large sample size, a retrospective analysis of a prospective design, and use of diverse predictive modeling approaches, which enhanced the robustness of the findings. Importantly, the reliance on CT—a widely available, cost-effective, and fast imaging modality—enhances the study's relevance to real-world clinical practice, particularly in LMIC, where 70% of strokes occur (18). Despite CT's limitations in assessing small vessel disease compared to MRI, this study demonstrates that with expert interpretation, CT can provide accurate and clinically relevant assessments of cSVD.

The mSVD score, as a simple composite of routinely available CT markers, may serve as a pragmatic tool for early risk stratification after thrombectomy. Its strong association with poor functional outcome could support clinicians in setting realistic expectations during early discussions with patients and families, and in planning post-acute care by identifying patients who may benefit from closer monitoring, intensive rehabilitation, or cognitive evaluation.

### Limitations

Several limitations should be acknowledged. The single-center design limits the generalizability of the findings, while the absence of randomization could result in residual confounding factors. Additionally, while CT is the most accessible imaging modality, it is not the gold standard for cSVD evaluation. Advanced imaging techniques, such as MRI, besides being less available, especially in LMIC, and more expensive, provide superior resolution and may offer valuable insights into the microvascular pathology underlying cSVD, allowing for the identification of several described markers (e.g., cerebral microbleeds and dilation of perivascular space) and facilitating easier evaluation of these conditions (19, 37). Furthermore, between the period of the cohort (2018–2022), the indications of MT were widened, and changes in guidelines occurred. However, this study shows that CT-based evaluations, when performed by trained specialists, can deliver actionable and accurate results, but the learning curve for the analysis must also be taken into consideration.

### Perspectives

Future research should explore whether cSVD markers and composite scores act as effect modifiers for thrombectomy outcomes, helping to determine whether patients with severe cSVD derive less benefit due to underlying microvascular impairment. External validation of the mSVD score in multicenter or population-based cohorts is also needed to confirm its generalizability across different clinical settings. In parallel, there is growing potential to leverage artificial intelligence to automate and standardize the assessment of cSVD markers on CT, such as leukoaraiosis (38). While this study does not support excluding patients from thrombectomy based on cSVD, it raises important questions about thresholds of brain resilience that may inform shared decision-making and individualized post-stroke care.

## Conclusions

This study demonstrates that composite scores derived from admission brain CT, particularly the modified Small Vessel Disease (mSVD) score, are powerful and independent predictors of poor functional outcome at 90 days after mechanical thrombectomy for anterior circulation large vessel occlusion. Among all variables tested—including clinical, radiological, and laboratory parameters—mSVD emerged as the most important predictor, outperforming individual small vessel disease markers and the Brain Frailty Score (BFS) in both traditional statistical models and machine learning analysis.

These findings highlight the clinical utility of a simple, accessible CT-based marker of cerebrovascular burden that captures brain frailty beyond what conventional predictors like age or NIHSS can reflect. The ability of mSVD to stratify prognosis and estimate the probability of functional outcomes underscores its potential role in refining thrombectomy decision-making and tailoring post-stroke care.

This is the first study to systematically compare composite cSVD scores against individual imaging markers using both regression and machine learning, establishing mSVD as a superior prognostic tool. Future studies should aim to validate these findings across broader populations and investigate the potential role of cSVD as an effect modifier in endovascular treatment outcomes. Such insights may help clinicians personalize care, optimize resource allocation, and improve outcomes for patients with substantial microvascular disease burden.

## Data Availability

Data supporting the findings of this study are available upon reasonable request to the corresponding author. Access will be granted to qualified researchers following the approval of a formal research protocol by the Institutional Review Board and the execution of a data use agreement.
