# Supplementary material for: Modified small vessel disease score as the top predictor of stroke outcome after thrombectomy: a CT-based machine learning study
Source: Front Neurol. 2026 Jun 23;17:1622586. doi: 10.3389/fneur.2026.1622586 (PMC13337418; doi:10.3389/fneur.2026.1622586)
Supplement: Supplementary file 1 [file Supplementary_file_1.docx]

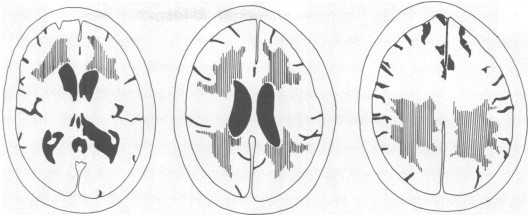


**Supplementary Figure 1.**
Assessment of leukoaraiosis on three standard axial CT slices: through the choroid plexus, the cella media, and the centrum semiovale.
Adapted from van Swieten et al. (1990).


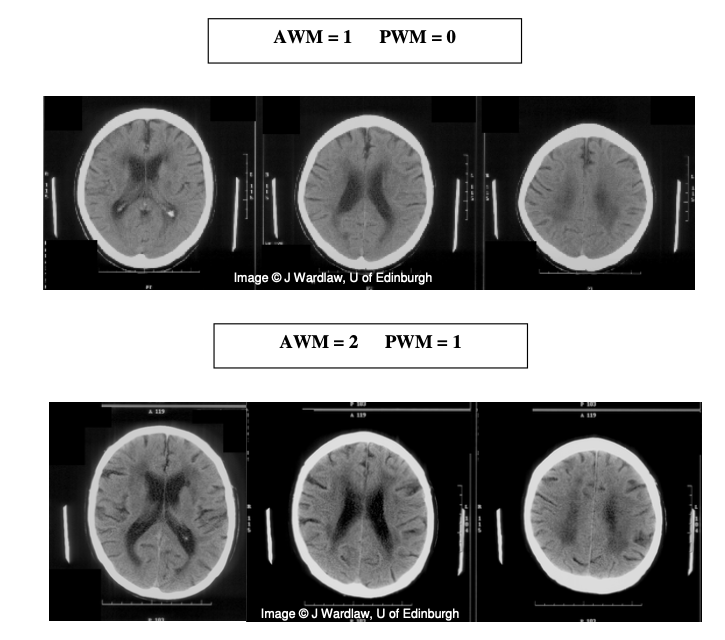


**Supplementary Figure 2.**
Examples of leukoaraiosis (white matter hypodensities) on CT using the van Swieten scale.
AWM: Anterior white matter score; PWM: Posterior white matter score.
Top row: Mild leukoaraiosis (AWM = 1; PWM = 0).
Bottom row: Severe leukoaraiosis (AWM = 2; PWM = 1).
Adapted from Wardlaw (n.d.)


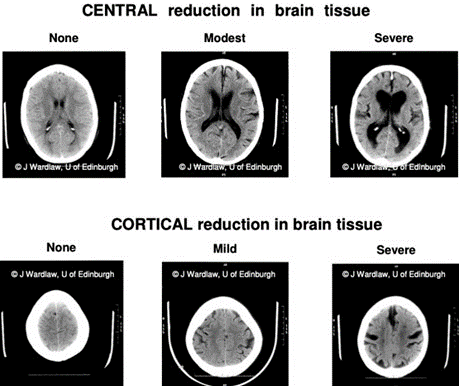


**Supplementary Figure 3.**
Assessment of brain atrophy on CT.
Top row: Central atrophy characterized by ventricular enlargement, graded as none, modest, or severe.
Bottom row: Cortical atrophy characterized by sulcal widening, also graded as none, mild, or severe.
Adapted from Wardlaw (n.d.)


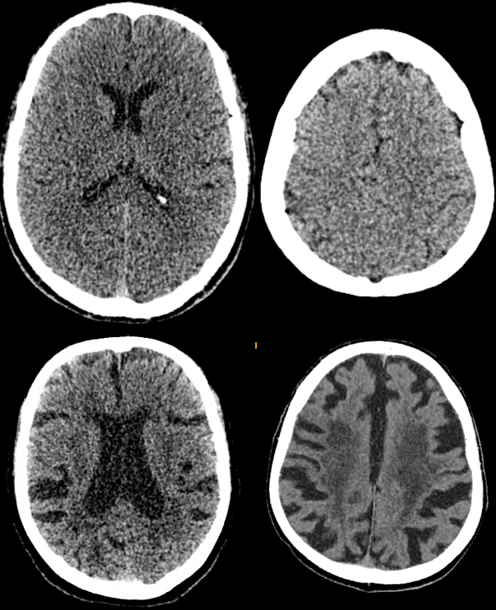


**Supplementary Figure 4.**
Representative CT scans from the study cohort illustrating the spectrum of CSVD burden.
**Top row:** Normal imaging, no evidence of leukoaraiosis or brain atrophy.
**Bottom row:** Severe leukoaraiosis (diffuse white matter hypodensities) and marked central and cortical brain atrophy.
These examples highlight the contrast between minimal and advanced CSVD burden as assessed using the CSVD scores.

**REFERENCES:**

‌‌Van Swieten, J C, A Hijdra, P J Koudstaal, and J van Gijn. 1990. “Grading White Matter Lesions on CT and MRI: A Simple Scale.” *Journal of Neurology, Neurosurgery & Psychiatry* 53 (12): 1080–83. https://doi.org/10.1136/jnnp.53.12.1080.

Wardlaw, J M “Acute Ischaemic Stroke Acute Ischaemic Stroke Acute Ischaemic Stroke Acute Ischaemic Stroke CT or MR SCAN READING FORM.” n.d. <https://www.ed.ac.uk/files/imports/fileManager/CT%20and%20MR%20reading%20form.pdf>.
