# Supplementary material for: Modified small vessel disease score as the top predictor of stroke outcome after thrombectomy: a CT-based machine learning study
Source: Front Neurol. 2026 Jun 23;17:1622586. doi: 10.3389/fneur.2026.1622586 (PMC13337418; doi:10.3389/fneur.2026.1622586)
Supplement: Supplementary file 2 [file Table_1.docx]

**Supplementary Table 1. Hosmer-Lemeshow Goodness-of-Fit and Brier Scores for Logistic Regression Models**.

| **Model** | **HL Chi-square** | **HL df** | **HL p-value** | **Brier Score** |
| --- | --- | --- | --- | --- |
| Model 1 | 8,93 | 8 | 0,348 | 0,121 |
| Model 2 | 9,34 | 8 | 0,315 | 0,125 |
| Model 3 | 5,58 | 8 | 0,694 | 0,129 |
| Model 4 | 4,74 | 8 | 0,785 | 0,138 |
| Model 5 | 5,22 | 8 | 0,734 | 0,120 |
| Model 6 | 3,30 | 8 | 0,914 | 0,123 |
| Model 7 | 14,50 | 8 | 0,070 | 0,125 |
| Model 8 | 14,81 | 8 | 0,063 | 0,078 |

Legend: HL – Hosmer-Lemeshow test, df – degrees of freedom
