# Supplementary material for: Modified small vessel disease score as the top predictor of stroke outcome after thrombectomy: a CT-based machine learning study
Source: Front Neurol. 2026 Jun 23;17:1622586. doi: 10.3389/fneur.2026.1622586 (PMC13337418; doi:10.3389/fneur.2026.1622586)
Supplement: Supplementary file 3 [file Data_Sheet_1.pdf]

Supplementary Table 1. Hosmer-Lemeshow Goodness-of-Fit and Brier Scores for Logistic Regression Models.

Model	HL Chi-square	HL df	HL p-value	Brier Score
Model 1	4.95	6	0.55	0.137
Model 2	7.39	6	0.286	0.139
Model 3	6.67	5	0.247	0.131
Model 4	4.06	4	0.397	0.129
Model 5	8.27	3	0.041	0.128
Model 6	6.89	2	0.032	0.127
Model 7	14.92	1	0	0.127
Model 8	9.99	-2		0.105

Legend: HL – Hosmer-Lemeshow test

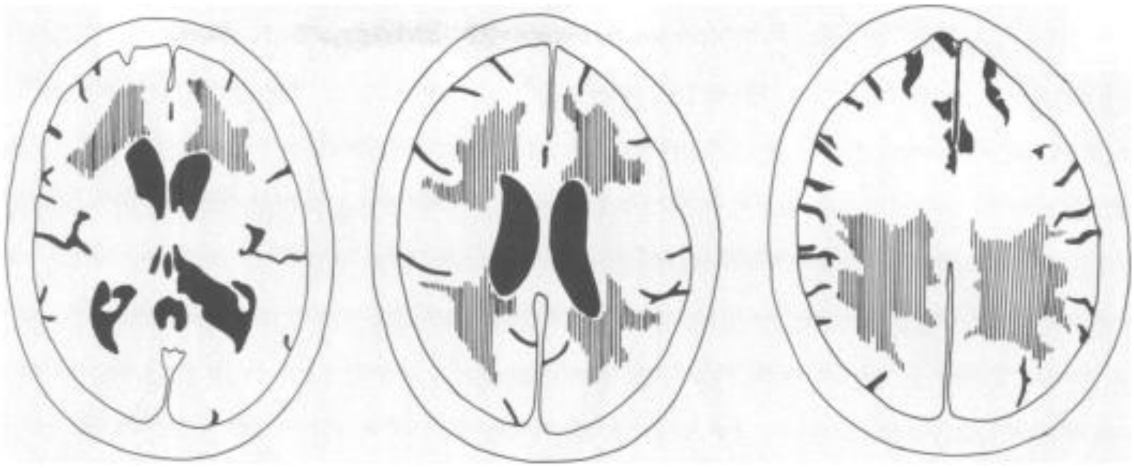

Supplementary Figure 1.

Assessment of leukoaraiosis on three standard axial CT slices: through the choroid plexus, the cella media, and the centrum semiovale.

Adapted from van Swieten et al. (1990).

AWM = 1   PWM = 0

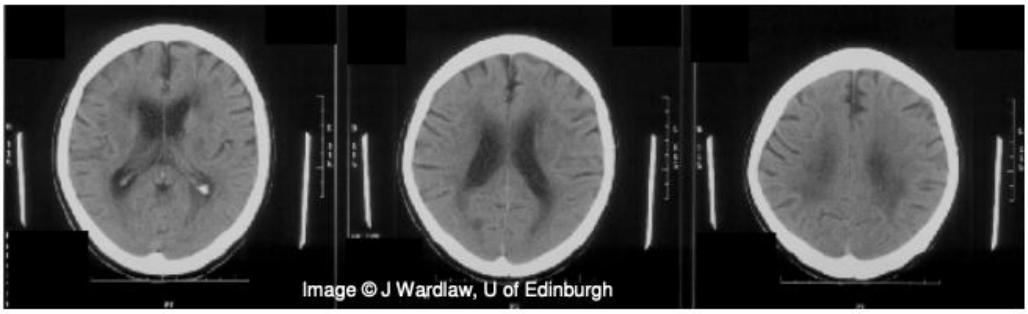

AWM = 2   PWM = 1

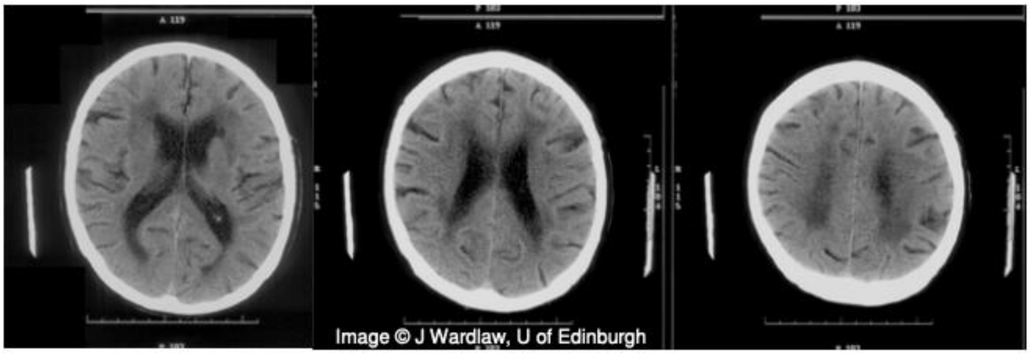

Supplementary Figure 2.
Examples of leukoaraiosis (white matter hypodensities) on CT using the van Swieten scale.
AWM: Anterior white matter score; PWM: Posterior white matter score.
Top row: Mild leukoaraiosis (AWM = 1; PWM = 0).
Bottom row: Severe leukoaraiosis (AWM = 2; PWM = 1).
Adapted from Wardlaw (n.d.)

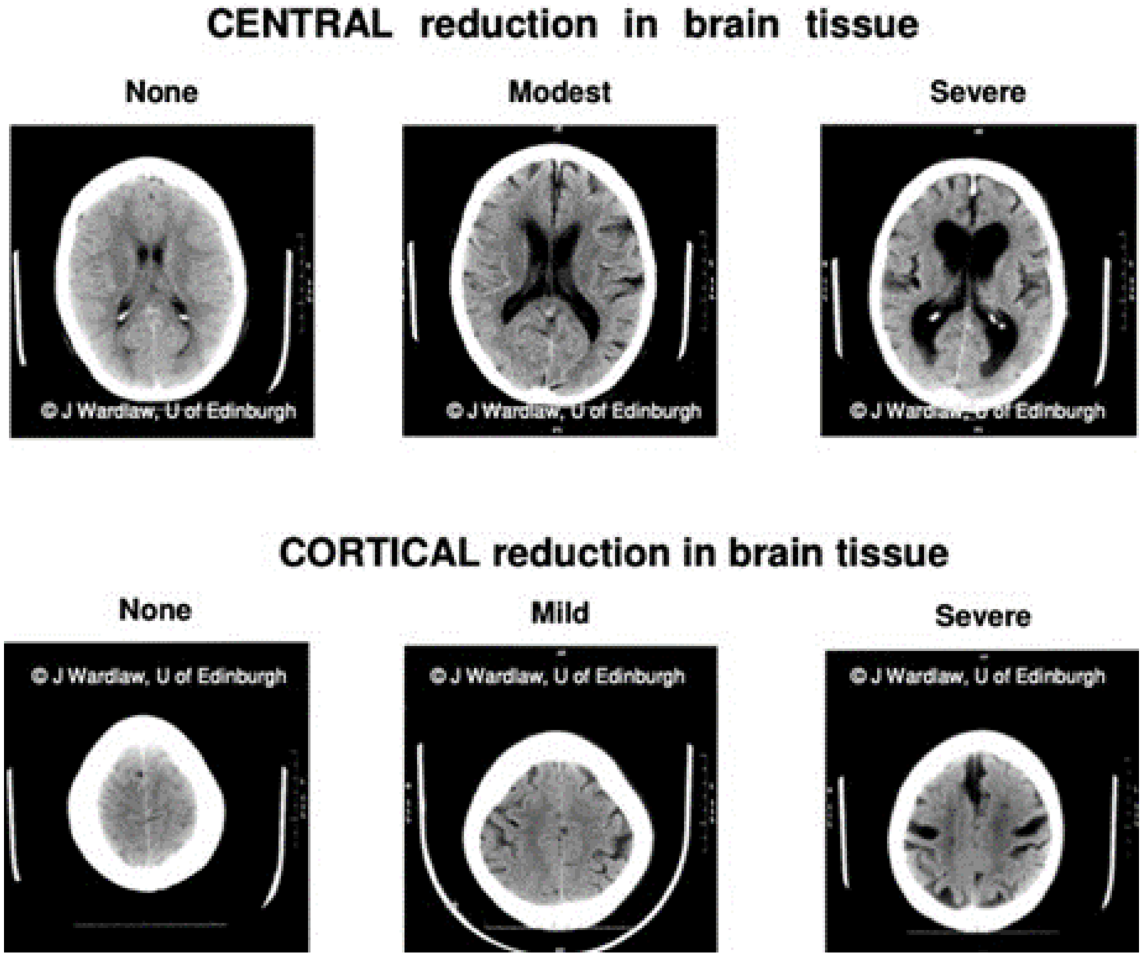

Supplementary Figure 3.
Assessment of brain atrophy on CT.
Top row: Central atrophy characterized by ventricular enlargement, graded as none, modest, or severe.
Bottom row: Cortical atrophy characterized by sulcal widening, also graded as none, mild, or severe.
Adapted from Wardlaw (n.d.)

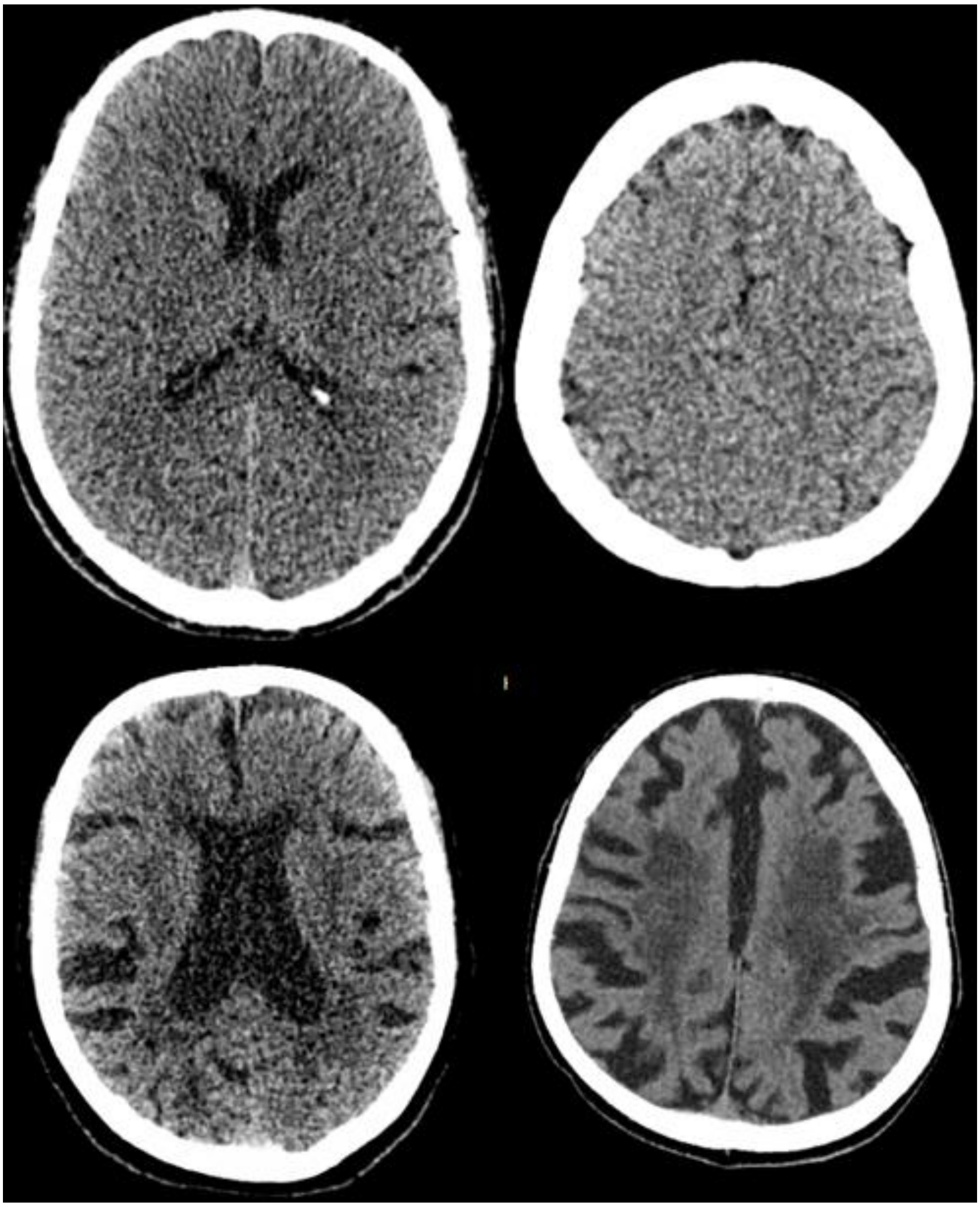

Supplementary Figure 4.
Representative CT scans from the study cohort illustrating the spectrum of CSVD burden.
Top row: Normal imaging, no evidence of leukoaraiosis or brain atrophy.
Bottom row: Severe leukoaraiosis (diffuse white matter hypodensities) and marked central and cortical brain atrophy.
These examples highlight the contrast between minimal and advanced CSVD burden as assessed using the CSVD scores.

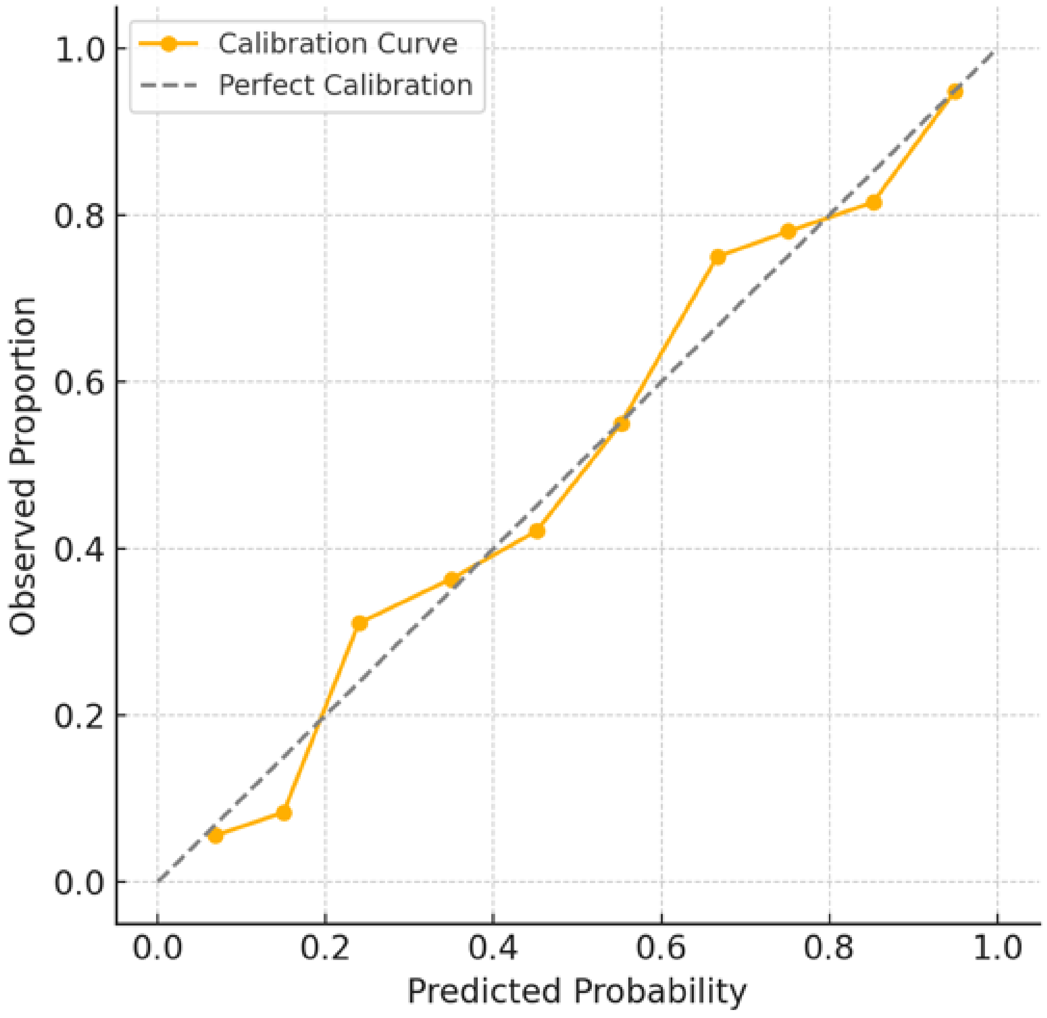

Supplementary Figure 5. Calibration Plot for Model 1 (mSVD Score Only Model)
The calibration curve compares predicted probabilities of 90-day functional dependence (mRS 3–6) against the observed proportions across deciles of risk. The dashed diagonal line indicates perfect calibration. Model 1 showed good calibration with a Hosmer-Lemeshow p-value of 0.55 and a Brier score of 0.137.

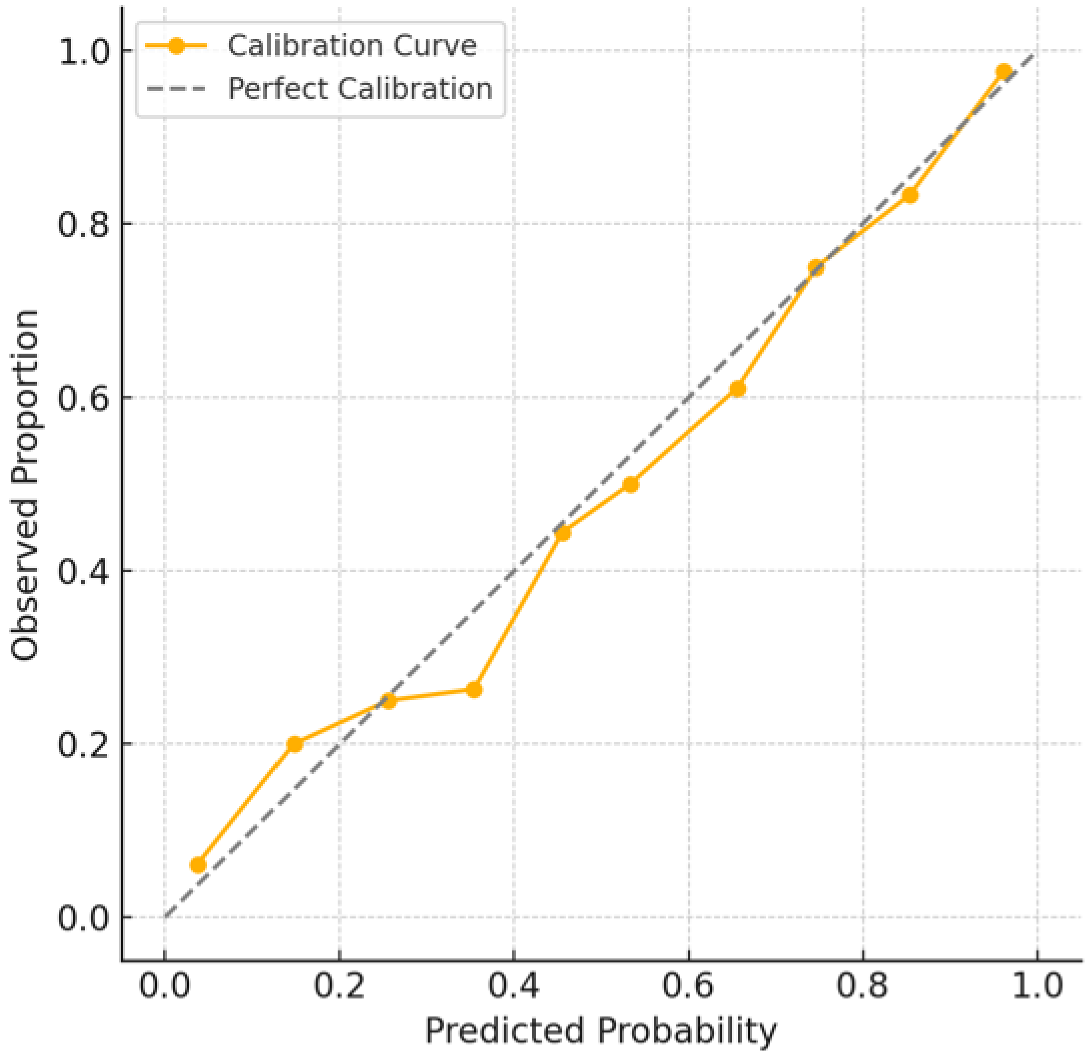

Supplementary Figure 6. Calibration Plot for Model 8 (Full Model)
This plot illustrates the agreement between predicted and observed probabilities for the full multivariable logistic regression model (Model 8). Despite a slightly wider deviation from the ideal line at the lowest probability deciles, the model retained reasonable overall calibration with a Brier score of 0.105. Hosmer-Lemeshow p-value could not be computed due to insufficient decile groups in high prediction bins.

REFERENCES:

Van Swieten, J C, A Hijdra, P J Koudstaal, and J van Gijn. 1990. "Grading White Matter Lesions on CT and MRI: A Simple Scale." *Journal of Neurology, Neurosurgery & Psychiatry* 53 (12): 1080–83. https://doi.org/10.1136/jnnp.53.12.1080.

Wardlaw, J M "Acute Ischaemic Stroke Acute Ischaemic Stroke Acute Ischaemic Stroke Acute Ischaemic Stroke CT or MR SCAN READING FORM." n.d. https://www.ed.ac.uk/files/imports/fileManager/CT%20and%20MR%20reading%20form.pdf.
